# Fiber‐Spinning‐Chemistry Method toward In Situ Generation of Highly Stable Halide Perovskite Nanocrystals

**DOI:** 10.1002/advs.201901694

**Published:** 2019-09-16

**Authors:** Xuan Lu, Yang Hu, Jiazhuang Guo, Cai‐Feng Wang, Su Chen

**Affiliations:** ^1^ State Key Laboratory of Materials‐Oriented Chemical Engineering College of Chemical Engineering, and Jiangsu Key Laboratory of Fine Chemicals and Functional Polymer Materials Nanjing Tech University Nanjing 210009 China

**Keywords:** electro‐microfluidic spinning, fiber spinning chemistry, perovskite nanocrystals, stability, water resistance

## Abstract

All‐inorganic halide perovskite nanocrystals (PNCs) have drawn increasing attention owing to their splendid optical properties. However, such nanomaterials suffer from intrinsic instability, greatly limiting their practical application. Meanwhile, environmental regulation has restricted the emissions of volatile organic compounds (VOCs), initiating a search for alternative approaches to PNC synthesis and film forming. Herein, fiber‐spinning chemistry (FSC) is proposed for easy‐to‐perform synthesis of highly stable PNC fibrous films. The FSC process utilizes spinning fibers as reactors, reducing the generation of VOCs. This method enables the fabrication of CsPbX_3_ (X = Cl, Br, I) PNCs/poly(methyl methacrylate)/thermoplastic polyurethanes fibrous films at room temperature in one step, exhibiting tunable emission between 450 and 660 nm. Significantly, the in situ generation of PNCs in hydrophobic core–shell nanofibers results in highly improved fluorescence stability. PNCs/polymer fibrous films keep constant in photoluminescence (PL) after storage at atmosphere for 90 d and retain 82% PL after water immersion for 120 h (vs fluorescence quenching in 10 d in air or 5 h in water for pristine PNCs). The PNCs/polymer fibrous films endowed with superior optical stability and great flexibility show promising potentials in flexible optoelectronic applications. This work paves a facile way toward high‐performance nanoparticles/polymer fibrous films.

## Graphical abstract

**Fiber spinning chemistry (FSC)** is proposed for easy‐to‐perform synthesis of perovskite nanocrystals (PNCs)/polymer fibrous films in one step. The FSC strategy greatly reduces the generation of volatile organic compounds. The as‐prepared PNCs/polymer fibrous films have tunable emission over nearly full visible spectrum, excellent air/water stability, and great flexibility, which are potentially useful for flexible optoelectronic applications.

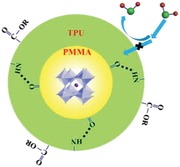

## 1 Introduction

Colloid perovskite nanocrystals (PNCs) have been developed as well‐known optical materials with high photoluminescence (PL) quantum yield (QY), adjustable fluorescence wavelength, and narrow full width at half maximum (FWHM).^1^ Such nanomaterials have aroused widespread attention in a variety of applications, such as light emitting diodes (LEDs),^2^ light‐emitting transistors,^3^ solar concentrators,^4^ solar cells,^5^ photodetectors,^6^ and next‐generation display devices.^7^ In particular, many approaches have been achieved to the handy preparation of all‐inorganic halide PNCs including CsPbX_3_ (X = Cl, Br, I) behaving full‐visible‐spectrum emission.^8^

Nevertheless, an unavoidable challenge remains to improve the stability of PNCs under atmosphere, humidity environment, and UV radiation.^9^ To this end, considerable endeavors have been devoted to the introduction of mixed cations allowing ionic radii satisfying the Goldschimdt's tolerance factor *t* closer to 1.^10^, ^11^ For instance, Ho‐Baillie and co‐workers utilized bulk organic ammonium iodide (iso‐butylammonium iodide) and formamidinium iodide to passivate perovskite, to preserve 87% initial performance after storage at ambient condition for 38 d.^12^ Besides, PNCs have been encapsulated in inorganic salts, nanosilica, and polymers, which can tighten the connection between the ligand and PNC surfaces, resulting in the enhanced stability of PNCs.^13^, ^14^, ^15^, ^16^, ^17^ Lin's group employed CaF_2_ as hierarchical matrices, preserving 60% of PL after 1 d storage in water.^15^ Yang's group encapsulated PNCs with polystyrene (PS) via electrospinning technique, to withhold 70% of initial PL QY after immersed in water for 192 h.^17^ Although these efforts have made a great progress on this issue, alternative methods are still highly needed to prepare PNCs with enhanced stability, especially those overcoming complicated procedures. Meanwhile, environmental regulation has restricted the emissions of volatile organic compounds (VOCs).^18^ However, large quantities of organic solvents have still been used in the synthesis of PNCs and their film‐forming process in most cases, which does not conform to the current environmentally friendly and sustainable concept. As a consequence, there is a growing demand for the preparation of stable PNCs film in an easy‐to‐perform and green way.

Herein, we demonstrate a facile method, named fiber‐spinning chemistry (FSC), enabling one‐step preparation of highly stable halide perovskite fibrous films with excellent air/water resistance. FSC we propose here means utilizing spinning fibers as reactors to carry out chemical reactions. Depending on spinning conditions, millimeter‐scale, microscale, and nanoscale reactors could be provided.^19^ This process not only circumvents the use of quantities of organic solvents, greatly reducing VOC generation, but also allows fiber substrates to serve as stabilizing ligands to tether nanoparticles in situ, improving the stability of nanoparticles. In this case, we develop FSC in nanoscale reactors established by core–shell polymeric nanofibers, to realize one‐step fabrication of highly stable, water‐resistant, and greatly flexible PNCs/polymer fibrous films at room temperature. This easy‐to‐perform and one‐step FSC process for preparation of PNCs/polymer films takes advantage over the common procedures usually involving synthesis of PNCs, incorporation into polymeric hosts, and film forming, and also reduces about 40% of waste output compared with spin‐coating method.^20^ The as‐prepared PNCs/polymer fibrous films show tunable PL emission ranging from 450 to 660 nm with narrow FWHM of 18–35 nm. More importantly, compared to the pristine PNCs showing complete PL quenching after storage under ambient condition for 10 d or immersed in water for 5 h, PNCs/polymer nanofiber films show almost no change in PL after storage at atmosphere for 90 d and preserve 82% of initial PL after immersed in water for 120 h, which surpass previously reported PNC composites in stability. Furthermore, with the merit of superior optical stability and great flexibility, such fibrous films are potentially useful in flexible or wearable optoelectronic applications. As a proof‐of‐concept experiment, compared with the spin‐coating method, the present work demonstrates an available route suitable to prepare various stable flexible polymer‐encapsulated PNCs and other nanoparticles with versatile functions and applications.

## 2 Results and Discussion

**Figure 1:**
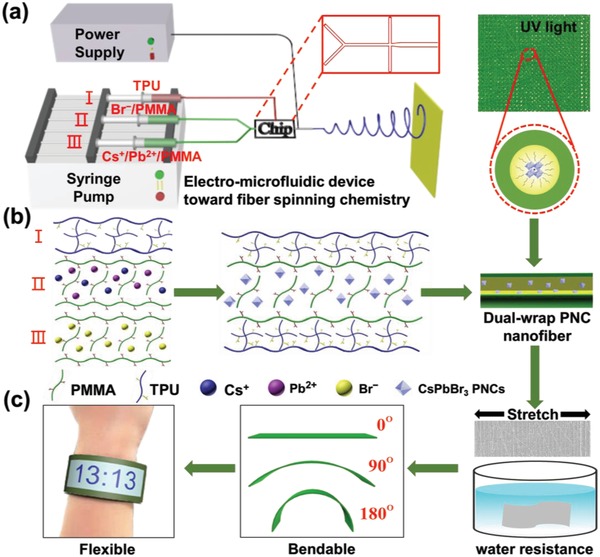
Schematic illustrations for fabrication of CsPbBr_3_–PMMA/TPU fibrous film toward flexible optoelectronic application. a) One‐step preparation of CsPbBr_3_/PMMA/TPU core–shell nanofiber films via electro‐microfluidic spinning technique. b) In situ formation mechanism of CsPbBr_3_/PMMA/TPU fibrous film via FSC strategy. c) Flexible and wearable optoelectronic device constructed from CsPbBr_3_/PMMA/TPU fibrous film with good water resistance.

In this paper, we employed an electro‐microfluidic spinning technique^21^ to implement FSC allowing one‐step rapid preparation of highly stable PNCs/polymer fibrous films at room temperature. In our previous work, PNC/poly(methyl methacrylate) (PMMA) nanocomposites were fabricated at junctions formed between microfluidic‐spinning microfibers and a spin‐coating layer, to maintain 75% PL after storage in air for 3 d.[qv: 19c] Here, core–shell nanofiber reactors were constructed via electro‐microfluidic spinning technique to carry out FSC in ambient air at room temperature, yielding PNCs/polymer fibrous films which keep constant in PL after storage at atmosphere for 90 d and retain 82% PL after immersed in water for 120 h. PMMA was chosen as core material owing to its high transparency up to 92%, excellent mechanical property, and good compatibility with precursor solutions of CsPbBr_3_. Similarly, thermoplastic polyurethane (TPU) was utilized as shell material owing to its good antioxidation and water resistance especially moisture stability. **Figure** **1**a,b shows a typical example for the fabrication of CsPbBr_3_/PMMA/TPU nanofiber from electro‐microfluidic FSC strategy. We designed a three‐fluid coaxial electro‐microfluidic spinning procedure. In this procedure, the Br^−^/PMMA fluid meeting with the Cs^+^/Pb^2+^/PMMA fluid at the Y‐shaped inlet of the chip played as the inner phase while the TPU flow acted as the outer phase, leading to the in situ generation of CsPbBr_3_ PNCs in PMMA/TPU core–shell nanofibers. Such dual‐wrap perovskite nanofibers were then collected on a receiver to form fibrous films. Interestingly, the as‐prepared fibrous films show good optical properties, high stability, water resistance, and great flexibility, and hence their potential applications in flexible/wearable optoelectronic devices were also demonstrated (Figure 1c). By varying Cl^−^/Br^−^/I^−^ ratios in the core precursor solutions, PNCs/PMMA/TPU fibrous films with tunable PL emission ranging from 450 to 660 nm were prepared, as listed in **Table** **1**. As such, we presented a simple approach enabling the in situ generation of various fluorescent PNCs/polymer fibrous films with high stability potentially useful for optoelectronic applications.

**Table 1:** Characteristics of PNCs/PMMA/TPU fibrous films with different compositions

Sample	PL emission peak [nm]	PL QY [%]	PL FWHM [nm]	Diameter [nm]
CsPb(Br*_x_*Cl_1−_ *_x_*)_3_/PMMA/TPU				
*X* = 0	460	7.8	19	253 ± 60
*X* = 0.3	485	8.9	21	292 ± 90
*X* = 0.7	505	9.9	24	353 ± 60
CsPbBr_3_				
CsPbBr_3_/PMMA/TPU	524	39	23	253 ± 60
Pure CsPbBr_3_ PNCs	520	42.3	21	–
CsPb(Br*_x_*I_1−_ *_x_*)_3_/PMMA/TPU				
*X* = 0	657	14.1	56	503 ± 75
*X* = 0.3	616	18.5	54	515 ± 80
*X* = 0.5	583	21.2	48	413 ± 100
*X* = 0.7	551	25.2	28	350 ± 60

**Figure 2:**
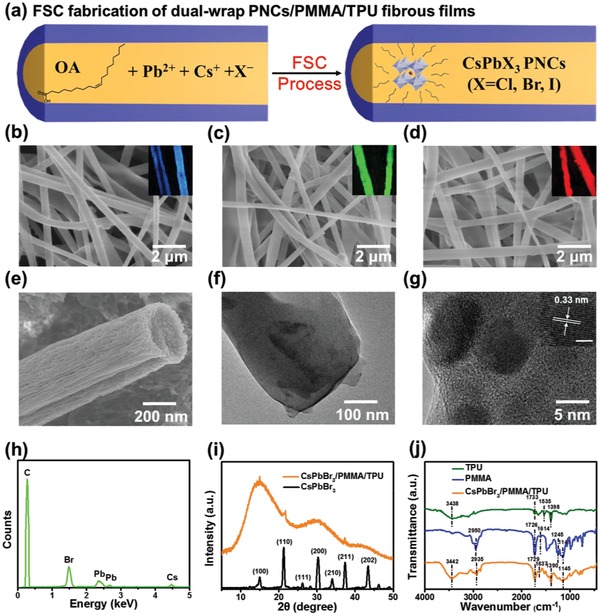
a) In situ generation of PNCs in PMMA/TPU fibrous films through electro‐microfluidic FSC process. SEM images of b) CsPb(Br_0.3_Cl_0.7_)_3_/PMMA/TPU, c) CsPbBr_3_/PMMA/TPU, and d) CsPb(Br_0.3_I_0.7_)_3_/PMMA/TPU fibrous films. Inset: Fluorescence confocal microscopy images of the corresponding samples. e) SEM image of a single CsPbBr_3_/PMMA/TPU nanofiber. f) TEM image and g) HRTEM image of CsPbBr_3_/PMMA/TPU nanofiber. Inset: HRTEM image of a single dot and the scale bar is 2 nm. h) EDX pattern of CsPbBr_3_/PMMA/TPU fibrous film. i) XRD patterns for CsPbBr_3_/PMMA/TPU fibrous film and pure CsPbBr_3_ PNCs. j) FT‐IR spectra of TPU, PMMA, and CsPbBr_3_/PMMA/TPU fibrous film.

The morphology of PNCs/PMMA/TPU fibrous films was investigated. As schematically displayed in **Figure** **2**a, PNCs are in situ generated in PMMA/TPU nanofibers to give PNCs/PMMA/TPU fibrous films through electro‐microfluidic FSC process. Scanning electron microscopy (SEM) images indicate that the fibrous films are composed of uniform fibers with diameters of hundreds of nanometers (Figure 2b–e and Table 1). The fiber diameter is dependent on various parameters such as voltage, solution concentration, flow rate, temperature, humidity, and uniformity of the solution during the spinning process.^22^ Fluorescence confocal microscopy confirms the fibrous morphology and verifies the fluorescence properties in these fibrous films (Figure 2b–d, inset). All of the nanofibers exhibit bright fluorescence, suggesting the generation of effective luminescent component PNCs and their good dispersion in the nanofibers. Figure 2f,g shows transmission electron microscopy (TEM) images of CsPbBr_3_/PMMA/TPU nanofibers. It can be clearly seen that PNCs with an average size of 9 nm are dispersed in the nanofiber (Figure 2f,g and Figure S1, Supporting Information). The high resolution transmission electron microscope (HRTEM) image testifies high crystallinity of PNCs with lattice fringes measured to be 0.33 nm (Figure 2g). These features reveal the feasibility of in situ generation of PNCs in polymer nanofiber via the one‐step electro‐microfluidic FSC process.

To further investigate the structure and chemical identity of PNCs/PMMA/TPU fibrous films, we performed energy dispersive X‐ray spectroscopy (EDX), X‐ray diffraction (XRD), and Fourier transform infrared (FT‐IR) spectroscopy. EDX spectroscopy reveals the elemental composition of CsPbBr_3_/PMMA/TPU fibrous film. As shown in Figure 2h, the elemental ratio of Cs:Pb:Br is ≈1:1:3, and the detection of a large amount of C elements is ascribed to the presence of the polymer matrices. Figure 2i shows XRD patterns of CsPbBr_3_/PMMA/TPU fibrous film and pure CsPbBr_3_ PNCs. In the XRD pattern of the film, the broadening of diffraction peaks is due to the presence of amorphous polymer matrices, while a series of relatively obvious crystallization peaks (100), (110), (200), (222), (220) of CsPbBr_3_ could be observed, indicating successful synthesis of PNCs/polymer. Figure 2j shows FT‐IR spectra of TPU, PMMA, and CsPbBr_3_/PMMA/TPU fibrous film. The FT‐IR spectrum of TPU demonstrates a symmetric stretching peak of NH at 3438 and 1535 cm^−1^, a distinct C=O related stretching vibration peak at 1733 cm^−1^, and a deformation vibration peak of C−O−C at 1078 cm^−1^. FT‐IR spectrum of PMMA manifests strong absorption peaks of methyl group and methylene group at 2950 and 2996 cm^−1^, and an ester carbonyl absorption peak at 1726 cm^−1^. Also at 1614 cm^−1^ is the absorption peak of the unreacted double bond in the polymer while at 1148 cm^−1^ an ester‐based characteristic peak belongs to PMMA.^23^ Most of the relevant characteristic peaks are present in the CsPbBr_3_/PMMA/TPU fibrous film.

**Figure 3:**
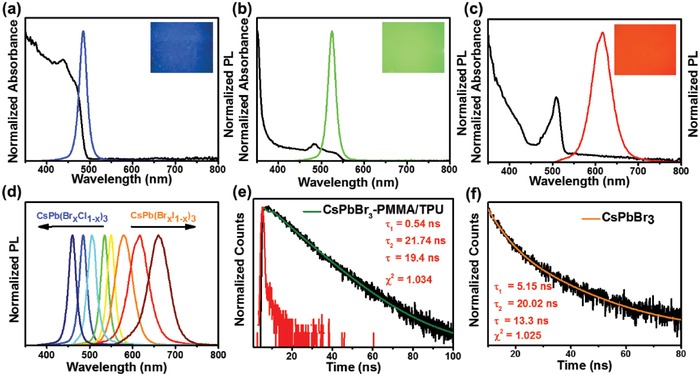
UV–vis absorption and emission spectra of a) CsPb(Br_0.3_Cl_0.7_)_3_/PMMA/TPU fibrous film, b) CsPbBr_3_/PMMA/TPU fibrous film, and c) CsPb(Br_0.3_I_0.7_)_3_/PMMA/TPU fibrous film. Inset: Photographs of the corresponding fibrous film under irradiation with a 365 nm UV light. d) PL emission spectra of nanofiber films with different fluorescence wavelengths. Time‐resolved fluorescence decay curves of e) CsPbBr_3_/PMMA/TPU fibrous film and instrument response function (IRF) (red) and f) CsPbBr_3_ PNC film. The data were fitted by the biexponential decay function (solid lines).

We utilized FSC method to prepare perovskite fibrous films with good optical properties and the large tunability in wavelength of fluorescence emission was realized (**Figure** **3**). The CsPbBr_3_/PMMA/TPU fibrous film has a strong absorption peak at 483 nm and the emission peak is sharp at 525 nm (Figure 3b and Figure S3, Supporting Information). To adjust the PL emission wavelength, mixed halogen PNC fibrous films, CsPb(Br*_x_*Cl_1−_ *_x_*)_3_/PMMA/TPU and CsPb(Br*_x_*I_1−_ *_x_*)_3_/PMMA/TPU, were synthesized through anion exchange in the electro‐microfluidic FSC process. Specifically, the CsPb(Br_0.3_Cl_0.7_)_3_/PMMA/TPU fibrous film shows a PL emission peak centered at 485 nm and UV–vis absorption at 438 nm (Figure 3a). Similarly, the CsPb(Br_0.3_I_0.7_)_3_/PMMA/TPU fibrous film possesses absorption peaks at 509 nm and emission peaks at 616 nm, respectively (Figure 3c). Correspondingly, the CsPb(Br_0.3_Cl_0.7_)_3_/PMMA/TPU, CsPbBr_3_/PMMA/TPU and CsPb(Br_0.3_I_0.7_)_3_/PMMA/TPU fibrous films exhibit blue, green, and red light under irradiation with ultraviolet light, respectively, and have narrow FWHM of 19, 23, and 54 nm, separately. The results indicate that the PNCs are uniform in size and well dispersed in the polymer. To prepare PNCs/PMMA/TPU nanofiber films with different fluorescence emission wavelengths, we changed the concentrations of dopant ions in the precursor solutions. The fluorescence wavelength can be adjusted from 460 to 660 nm (Table 1).

To better understand the effect of polymer coating on the luminescence of PNCs, time‐resolved fluorescence decay was performed (Figure 3e,f). The PL decay curve was fitted by biexponential decay function

(1) $$\mathrm{Fit}=A+B_{1}\exp\left(-\frac{t}{\tau_{1}}\right)\;\;+B_{2}\exp\left(-\frac{t}{\tau_{2}}\right)$$

As comparison, CsPbBr_3_ PNC solution was spin coated on PMMA substrate to make a film, termed pure CsPbBr_3_ film. The corresponding amplitude *B*_1_ (10.8%) of fast decay τ_1_ (0.54 ns) for CsPbBr_3_/PMMA/TPU fibrous film is much smaller than that (45.22%) of the fast decay τ_1_ (5.15 ns) for pure CsPbBr_3_ film (Table S1, Supporting Information). Thus, the trap‐assisted recombination from PNCs/polymer is much less compared with CsPbBr_3_.^24^ In addition, the slow decay τ_2_ of the CsPbBr_3_/PMMA/TPU fibrous film related to radiative recombination is 21.74 ns, larger than the value 20.02 ns for pure CsPbBr_3_ film. The increase in fluorescence lifetime could be due to the fact that the polymer matrices have a role in limiting the ion migration of the generated CsPbBr_3_, whilst well separating PNCs to decrease energy transfer among neighboring dots. In general, the value τ of CsPbBr_3_/PMMA/TPU fibrous film is 19.4 ns while that of pure CsPbBr_3_ film is 13.3 ns.

**Figure 4:**
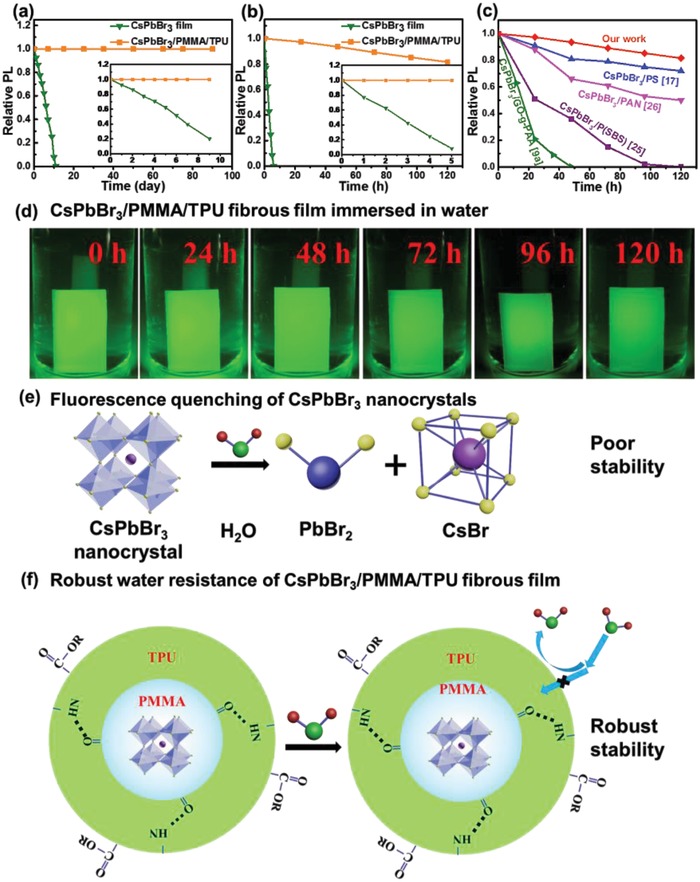
a) Stability test of CsPbBr_3_ film and CsPbBr_3_/PMMA/TPU film at 25 °C and 80% humidity. b) Stability test of CsPbBr_3_ film and CsPbBr_3_/PMMA/TPU film immersed in water. c) Comparison of water resistance of CsPbBr_3_ composites in this work with that in related reports. d) Photographs of permeated CsPbBr_3_/PMMA/TPU film in water for different period of time, taken under irradiation with a 365 nm UV light. e) Schematic illustration for fluorescence quenching of CsPbBr_3_ PNCs. f) Mechanism for robust water resistance of CsPbBr_3_/PMMA/TPU perovskite fibrous film.

It is well known that PNCs and their films are very sensitive to moisture and are susceptible to fluorescence quenching. Interestingly, PNCs/PMMA/TPU fibrous films synthesized via electro‐microfluidic FSC method herein have significantly improved air stability and water resistance (**Figure** **4**). In order to verify this, we performed long‐term stability tests on CsPbBr_3_ film and CsPbBr_3_/PMMA/TPU fibrous film under storage at 25 °C and 80% humidity. As shown in Figure 4a, the CsPbBr_3_/PMMA/TPU fibrous film has almost no change in fluorescence intensity after stored for 90 d, while the CsPbBr_3_ film shows complete fluorescence quenching in 10 d. Then we tested the stability of the fibrous film immersed in water. Figure 4b shows temporal evolution of PL intensity for CsPbBr_3_/PMMA/TPU fibrous film fully immersed in water, along with that of pure CsPbBr_3_ film for comparison. For the CsPbBr_3_/PMMA/TPU fibrous film, the PL intensity decreases slowly upon immersing time, and 82% of initial PL intensity remains after 120 h immersion. As a striking contrast, the PL intensity of CsPbBr_3_ film only remains less than 10% after 5 h and quenches completely in less than 10 h. Figure 4d shows photographs of the CsPbBr_3_/PMMA/TPU fibrous film immersed in water for different periods, which were taken under irradiation with a 365 nm UV light. We can see that even after soaking for 120 h, the film still emits bright green fluorescence. Recently, many efforts have been devoted to the incorporation of PNCs into the polymer matrices to improve the stability of PNCs in water. As shown in Figure 4c, CsPbBr_3_/polyacrylic acid‐grafted‐graphene oxide preserves 70% of initial PL intensity after immersed in water for 12 h,[qv: 9a] while 50% at 24 h for CsPbBr_3_/poly(styrene‐butadiene‐styrene),^25^ over 50% at 48 h for CsPbBr_3_/polyacrylonitrile (PAN),^26^ and 81% at 48 h for CsPbBr_3_/PS^17^ were realized. The CsPbBr_3_/PMMA/TPU fibrous film developed in this case, remaining 82% of initial PL intensity after storage in water for 120 h, shows superior water resistance. In addition, no obvious change in water stability of CsPbBr_3_/PMMA/TPU film was observed by varying CsPbBr_3_ content (Figure S4, Supporting Information). The effect of fiber diameter on PL performance of the CsPbBr_3_/PMMA/TPU film was also investigated. As shown in Figure S5 in the Supporting Information, as the fiber diameter increases, the measured PL intensity and PL QY decrease without obvious change in FWHM, and the water resistance gets further improved.

The origin of improved PL stability for PNCs/PMMA/TPU fibrous films is discussed. PNCs suffer from intrinsic instability and accelerated degradation occurs upon contact with water or moisture, resulting in the fluorescence quenching (Figure 4e).^27^ In this case, PNCs were in situ generated in PMMA/TPU polymeric nanofibers via FSC (Figure 2), and hydrogen bonding between TPU and the PMMA makes the core–shell architecture of PNCs/PMMA/TPU nanofibers more stable, and hence the inner PNCs are effectively stabilized by the outer polymers (Figure 4f). On one hand, the peripheral polymers well prevent the direct contact of PNCs with ambient oxygen and water, to effectively circumvent fluorescence quenching caused by the interaction with external stimuli. Specifically, CsPbBr_3_/PMMA/TPU fibrous film shows water contact angle of 101.82° while that of pure CsPbBr_3_ film is 77.95° (Figure S2, Supporting Information). The enhanced hydrophobicity of the fibrous film could efficiently block water outside. On the other hand, PNCs are well immobilized in the polymeric matrix, and hence the migration of ions could be sufficiently suppressed. Therefore, FSC‐developed PNCs/PMMA/TPU fibrous films show greatly improved air stability and water stability.

**Figure 5:**
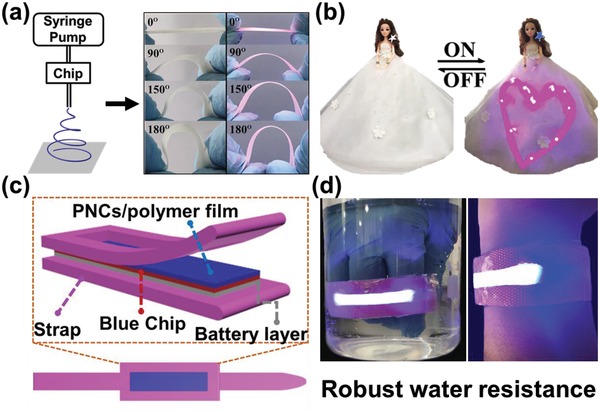
a) Schematic illustrations for fabrication of CsPbBr_3_/PMMA/TPU fibrous film (left) and the photographs of CsPb(Br_0.3_I_0.7_)_3_/PMMA/TPU fibrous film with bending angle of 0°, 90°, 150°, and 180° taken under daylight (middle) and UV light (right). b) Photographs of a heart‐shape LED and a star‐shape LED on a toy taken under OFF (left) and ON (right) states. c) Schematic structure of a conceptual flexible wearable illuminating bracelet. d) Photographs of the flexible wearable bracelet with robust water resistance.

Thanks to their good optical properties and high stability, PNCs/PMMA/TPU fibrous films show potential as color conversion materials to construct various LED devices. The fibrous films prepared by electro‐microfluidic FSC process appear outstanding flexibility (**Figure** **5**) and a certain mechanical strength (Figure S6, Supporting Information). As shown in Figure 5a, the as‐prepared CsPb(Br_0.3_I_0.7_)_3_/PMMA/TPU fibrous film can be easily bended at different angles of 0°, 90°, 150°, and 180° (Figure 5a). As such, flexible optoelectronic devices with desirable shapes could be achieved. For instance, we constructed different shapes of LED devices by covering PNCs/PMMA/TPU fibrous film on UV LED chips. As shown in Figure 5b, a heart‐shape LED and a star‐shape LED, constructed with use of CsPb(Br_0.3_I_0.7_)_3_/PMMA/TPU film and CsPb(Br_0.3_Cl_0.7_)_3_/PMMA/TPU film as color conversion material respectively, emit bright light at ON state. In view of the robust water resistance and bending performance, we also made use of this fibrous film to develop a wearable bracelet, which was constructed by using yellow‐emitting CsPb(Br_0.5_I_0.5_)_3_/PMMA/TPU fibrous film as color conversion materials covered on a GaN chip (Figure 5c). As shown in Figure 5d, the wearable bracelet emits bright white light and it works even when immersed in water. The results suggest the great potential of PNCs/PMMA/TPU fibrous films for flexible optoelectronic applications.

## 3 Conclusion

In summary, this work demonstrates an easy‐to‐perform route to prepare PNCs/polymer fibrous films useful for flexible optoelectronic applications. We developed FSC in nanoscale reactors established by electro‐microfluidic‐spun core–shell polymeric nanofibers, to realize one‐step fabrication of PNCs/PMMA/TPU fibrous films at room temperature. The as‐prepared PNCs/polymer fibrous films have tunable emission over nearly full visible spectrum, excellent air/water stability, and great flexibility. Specifically, PNCs/polymer fibrous films surpass previously reported PNC composites in stability; they keep unchanged in fluorescence properties after stored under ambient condition for 90 d and preserve 82% of initial PL after immersed in water for 120 h. Furthermore, we employed PNCs/PMMA/TPU fibrous films as color conversion materials to construct various flexible LED devices. We anticipate FSC strategy can be used universally as alternative green synthesis route for fabrication of various nanoparticles/polymer nanofiber films with high performance.

## 4 Experimental Section

*Materials*: All chemicals used in this work were commercially available and used without further purification. Lead oxide (PbO, 99.9%), barium acetate (C_2_H_3_CsO_2_, 99%), oleic acid (OA, 85%), oleylamine (OLA, 85%), tetraoctylammonium bromide (C_32_H_68_BrN, 98%), tetraoctylammonium chloride (C_32_H_68_ClN, 98%), tetraoctylammonium iodide (C_32_H_68_IN, 98%), dichloromethane (CH_2_Cl_2_, 99.5%), toluene (C_7_H_8_, AR), dimethylformamide (DMF, AR), and TPU extrusion grade (SU) were purchased from Aladdin Chemistry. PMMA SU was purchased from Tokyo Chemical Industry.

*Preparation of Precursor Solutions*: To prepare the Cs^+^/Pb^2+^/PMMA precursor solution, PbO (4 mmol), C_2_H_3_CsO_2_ (4 mmol), oleic acid (16 mL), and oleylamine (4 mL) were added into a 50 mL glass vessel. The mixture was heated at 150 °C under magnetic stirring until it became transparent. Then 20 mL of solvent mixture of dichloromethane and toluene (1:1 in volume ratio) and 4 g of PMMA were added into the mixture, which was stirred for 48 h to obtain the Cs^+^/Pb^2+^/PMMA precursor solution.

To prepare the Br^−^/PMMA precursor solution, tetraoctylammonium bromide (1.2 mmol), OA (3 mL), and OLA (1 mL) were added into 40 mL of solvent mixture of CH_2_Cl_2_ and toluene (1:1 in volume ratio) in a 50 mL glass vessel. The mixture was stirred until clarified, followed by the addition of 4 g of PMMA. The resultant mixture was stirred for 48 h to yield the Br^−^/PMMA precursor solution.2.5 g of TPU were dissolved in 50 mL of DMF by mechanical stirring at 60 °C for 48 h. The shell precursor solution was placed overnight to eliminate bubbles for further use.

*Synthesis of CsPbBr_3_/PMMA/TPU Fluorescent Fibrous Films* via *FSC*: The fabrication of CsPbBr_3_/PMMA/TPU fluorescent fibrous films was performed on an electro‐microfluidic spinning device (Nanjing Janus New‐Materials Co. Ltd). A three‐fluid coaxial electro‐microfluidic spinning mode was designed to fabricate the core‐shell fibrous film. Three syringes were used and each one was connected to a separate needle. Two needles having a diameter of 0.41 mm flowing through the core solution were attached to the Y‐shaped inlet of the chip, and a needle having a needle diameter of 0.84 mm flowing on the shell solution was on the side thereof. As such, a three‐fluid coaxial electro‐microfluidic system was obtained to fabricate CsPbBr_3_/PMMA/TPU fibrous film. The three precursor solutions were separately injected in 20 mL syringes which were placed in the syringe pump that fed continuously into the coaxial system (Figure 1a). The flow speed of the core and shell precursor solutions were 0.2 and 0.6 mL h^−1^, respectively. The tip of the liquid channel was connected to a power supply. The resulting solution was spun for 2 h at 500 rpm at a voltage of 20 kV and a spinning distance of 15 cm for collecting the nonwoven fibrous film. The electro‐microfluidic spinning process was manipulated at 24 °C and 80% relative humidity. Then, the collected fibrous film was dried at 70 °C for 3 h in vacuum oven to remove the residual DMF and toluene.

*Synthesis of Other Halide Perovskite/Polymer Fibrous Films* via *FSC*: Anion exchange was carried out to prepare PNCs/PMMA/TPU fibrous films with a widely tunable emission, which provide Cl anion and I anion from C_32_H_68_ClN and C_32_H_68_IN, respectively. To obtain CsPb(Br*_x_*Cl_1−_ *_x_*)_3_ and CsPb(Br*_x_*I_1−_ *_x_*)_3_, respectively, C_32_H_68_ClN and C_32_H_68_IN were added separately into the Br^−^/PMMA precursor solution to achieve ion doping. A precursor solution with different molar ratio of Br^−^:Cl^−^ or Br^−^:I^−^ was obtained by changing the concentrations of C_32_H_68_ClN or C_32_H_68_IN dissolving in dichloromethane/toluene mixture. Thereby, a series of PNCs/PMMA/TPU fibrous films could be prepared with adjustable fluorescence wavelength.

*Synthesis of Pure All‐Inorganic PNCs at Room Temperature*: A Cs^+^/Pb^2+^ precursor solution and a Br^−^ precursor solution were prepared as described above for the Cs^+^/Pb^2+^/PMMA precursor solution and the Br^−^/PMMA precursor solution, respectively, except no PMMA was added. 1 mL of Cs^+^/Pb^2+^ precursor solution and 1 mL of Br^−^ precursor solution was added into 10 mL of dichloromethane/toluene mixture (1:1 in volume ratio) with stirring. The mixture was stirred for 1 h, to provide the solution of CsPbBr_3_ PNCs. The PNC solution was spin coated on PMMA substrate to make a film, termed pure CsPbBr_3_ film.

*Application as Color‐Converting Film in the Flexible Wearable Bracelet*: A yellow‐emitting CsPb(Br_0.5_I_0.5_)_3_/PMMA/TPU fibrous film was placed on blue GaN chips to construct a wearable ring, which was then connected to a battery layer and sealed in a strap for research purpose.

*Characterization*: X‐ray diffraction (XRD) patterns were obtained from a D5005 X‐ray diffractometer (Siemens AG, Munich, Germany) from 5° to 80° at a scanning speed of 5° min^−1^. The morphology of the fibrous film was observed by SEM with a QUANTA 200 (Philips‐FEI, Holland) instrument at 20.0 keV. TEM images were taken with JEOL JEM‐2100 operated at 200 keV. The steady‐state PL spectra were collected by using a Varian Cary Eclipse spectrophotometer at room temperature. PL QY and time‐resolved PL were measured by Edinburgh FLS980. UV–vis absorption spectra were recorded on a Perkin‐Elmer Lambda 900 UV–vis spectrometer. Fluorescence images were measured on a laser scanning confocal microscope (Leica Company).

## Conflict of Interest

The authors declare no conflict of interest.

## Supporting information

Supplementary

## References

[1] Y. Wei , Z. Cheng , J. Lin , Chem. Soc. Rev. 2019, 48, 310. 30465675 10.1039/c8cs00740c

[2] a) L. N. Quan , F. P. Garcia de Arquer , R. P. Sabatini , E. H. Sargent , Adv. Mater. 2018, 30, e1801996; 30160805 10.1002/adma.201801996

[3] X. Y. Chin , D. Cortecchia , J. Yin , A. Bruno , C. Soci , Nat. Commun. 2015, 6, 7383. 26108967 10.1038/ncomms8383 PMC4491174

[4] a) H. G. Zhao , D. Benetti , X. Tong , H. Zhang , Y. F. Zhou , G. J. Liu , D. L. Ma , S. H. Sun , Z. M. M. Wang , Y. Q. Wang , F. Rosei , Nano Energy 2018, 50, 756;

[5] a) Y. Hou , X. Du , S. Scheiner , D. P. McMeekin , Z. Wang , N. Li , M. S. Killian , H. Chen , M. Richter , I. Levchuk , N. Schrenker , E. Spiecker , T. Stubhan , N. A. Luechinger , A. Hirsch , P. Schmuki , H. P. Steinruck , R. H. Fink , M. Halik , H. J. Snaith , C. J. Brabec , Science 2017, 358, 1192; 29123021 10.1126/science.aao5561

[6] a) F. Cao , L. Meng , M. Wang , W. Tian , L. Li , Adv. Mater. 2019, 31, e1806725; 30697825 10.1002/adma.201806725

[7] Y. L. Tong , Y. W. Zhang , K. Ma , R. Cheng , F. Wang , S. Chen , ACS Appl. Mater. Interfaces 2018, 10, 31603. 30152231 10.1021/acsami.8b10366

[8] L. Protesescu , S. Yakunin , M. I. Bodnarchuk , F. Krieg , R. Caputo , C. H. Hendon , R. X. Yang , A. Walsh , M. V. Kovalenko , Nano Lett. 2015, 15, 3692. 25633588 10.1021/nl5048779 PMC4462997

[9] a) A. Pan , M. J. Jurow , F. Qiu , J. Yang , B. Ren , J. J. Urban , L. He , Y. Liu , Nano Lett. 2017, 17, 6759; 28968132 10.1021/acs.nanolett.7b02959

[10] a) Y. Wu , P. Wang , X. Zhu , Q. Zhang , Z. Wang , Y. Liu , G. Zou , Y. Dai , M. H. Whangbo , B. Huang , Adv. Mater. 2018, 30, 1704324; 10.1002/adma.201704342 29315831

[11] L. Liang , L. Wencong , C. Nianyi , J. Phys. Chem. Solids 2004, 65, 855.

[12] Y. Y. Cho , A. M. Soufiani , J. S. Yun , J. C. Kim , D. S. Lee , J. Seidel , X. F. Deng , M. A. Green , S. J. Huang , A. W. Y. Ho‐Baillie , Adv. Energy Mater. 2018, 8, 1703392.

[13] F. Zhang , Z. F. Shi , Z. Z. Ma , Y. Li , S. Li , D. Wu , T. T. Xu , X. J. Li , C. X. Shan , G. T. Du , Nanoscale 2018, 10, 20131. 30376029 10.1039/c8nr07022a

[14] D. D. Yang , X. M. Li , H. B. Zeng , Adv. Mater. Interfaces 2018, 5, 1701662.

[15] Y. Wei , H. Xiao , Z. X. Xie , S. Liang , S. S. Liang , X. C. Cai , S. S. Huang , A. A. Al Kheraif , H. S. Jang , Z. Y. Cheng , J. Lin , Adv. Opt. Mater. 2018, 6, 1701343.

[16] W. Cha , H. J. Kim , S. Lee , J. Kim , J. Mater. Chem. C 2017, 5, 6667.

[17] H. Liao , S. Guo , S. Cao , L. Wang , F. Gao , Z. Yang , J. Zheng , W. Yang , Adv. Opt. Mater. 2018, 6, 1800346.

[18] D. Prat , J. Hayler , A. Wells , Green Chem. 2014, 16, 4546.

[19] a) L. L. Xu , C. F. Wang , S. Chen , Angew. Chem., Int. Ed. 2014, 53, 3988; 10.1002/anie.201310977 24595996

[20] a) Q. Zhou , Z. Bai , W. G. Lu , Y. Wang , B. Zou , H. Zhong , Adv. Mater. 2016, 28, 9163; 27571569 10.1002/adma.201602651

[21] a) G. Yang , X. Li , Y. He , J. Ma , G. Ni , S. Zhou , Prog. Polym. Sci. 2018, 81, 80;

[22] a) J. Xue , T. Wu , Y. Dai , Y. Xia , Chem. Rev. 2019, 119, 5298; 30916938 10.1021/acs.chemrev.8b00593 PMC6589095

[23] a) P. Zhang , X. Zhao , Y. Ji , Z. Ouyang , X. Wen , J. Li , Z. Su , G. Wei , J. Mater. Chem. B 2015, 3, 2487; 10.1039/c4tb02092h 32262123

[24] a) H. C. Cho , S. H. Jeong , M. H. Park , Y. H. Kim , C. Wolf , C. L. Lee , J. H. Heo , A. Sadhanala , N. Myoung , S. Yoo , S. H. Im , R. H. Friend , T. W. Lee , Science 2015, 350, 1222; 26785482 10.1126/science.aad1818

[25] C. C. Lin , D. H. Jiang , C. C. Kuo , C. J. Cho , Y. H. Tsai , T. Satoh , C. Su , ACS Appl. Mater. Interfaces 2018, 10, 2210. 29308867 10.1021/acsami.7b15989

[26] P. C. Tsai , J. Y. Chen , E. Ercan , C. C. Chueh , S. H. Tung , W. C. Chen , Small 2018, 14, 1704379. 10.1002/smll.201704379 29709108

[27] a) H. S. Kim , J. Y. Seo , N. G. Park , ChemSusChem 2016, 9, 2528; 27535474 10.1002/cssc.201600915

