# Supplementary material for: Fiber‐Spinning‐Chemistry Method toward In Situ Generation of Highly Stable Halide Perovskite Nanocrystals
Source: Adv Sci (Weinh). 2019 Sep 16;6(22):1901694. doi: 10.1002/advs.201901694 (PMC6864515; doi:10.1002/advs.201901694)
Supplement: Supplementary file 1 — Supplementary [file ADVS-6-1901694-s001.pdf]

Copyright WILEY-VCH Verlag GmbH & Co. KGaA, 69469 Weinheim, Germany, 2019.

ADVANCED SCIENCE
Open Access

Supporting Information

for *Adv. Sci.,* DOI: 10.1002/advs.201901694

Fiber-Spinning-Chemistry Method toward In Situ Generation of Highly Stable Halide Perovskite Nanocrystals

Xuan Lu, Yang Hu, Jiazhuang Guo, Cai-Feng Wang, and Su Chen**

Copyright WILEY-VCH Verlag GmbH & Co. KGaA, 69469 Weinheim, Germany, 2019.

Supporting Information

Fiber-Spinning-Chemistry Method towards In-Situ Generation of Highly Stable Halide Perovskite Nanocrystals

Xuan Lu, Yang Hu, Jiazhuang Guo, Cai-Feng Wang, and Su Chen**

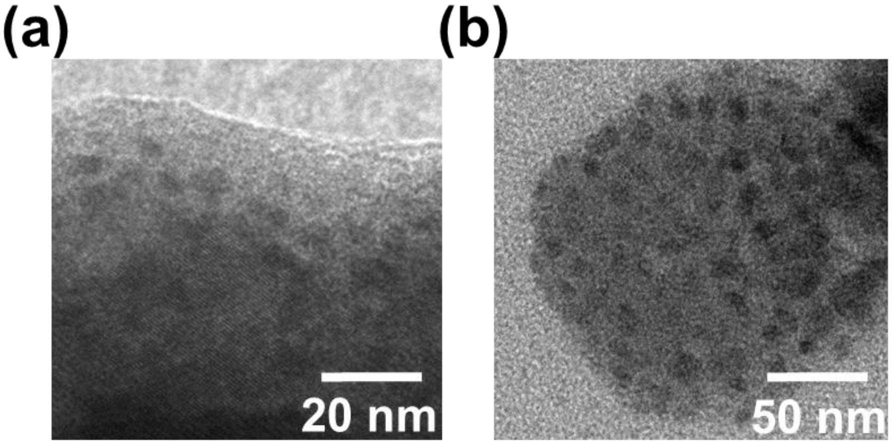

Figure S1. TEM images of PNCs in PMMA/TPU nanofibers. The sample was ultrasonically treated in ethanol for half an hour prior to TEM measurement.

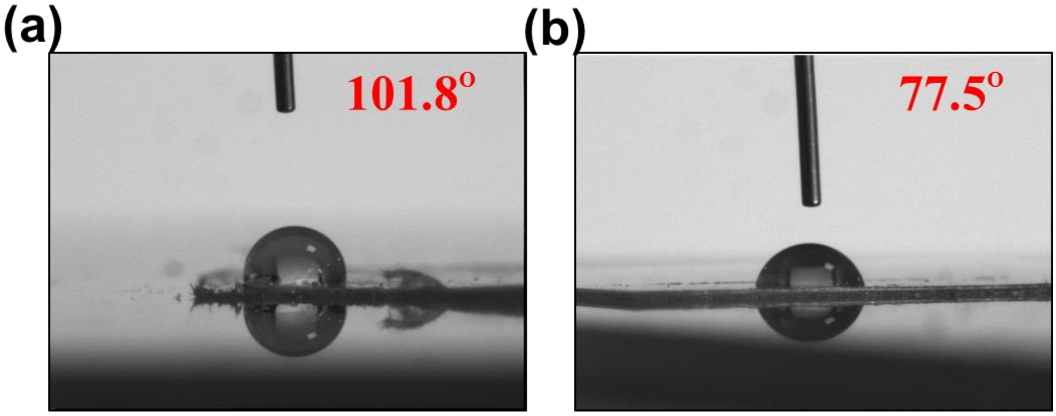

Figure S2. Water contact angle images of a) $CsPbBr_3$/PMMA/TPU fibrous film and b) pure $CsPbBr_3$ film.

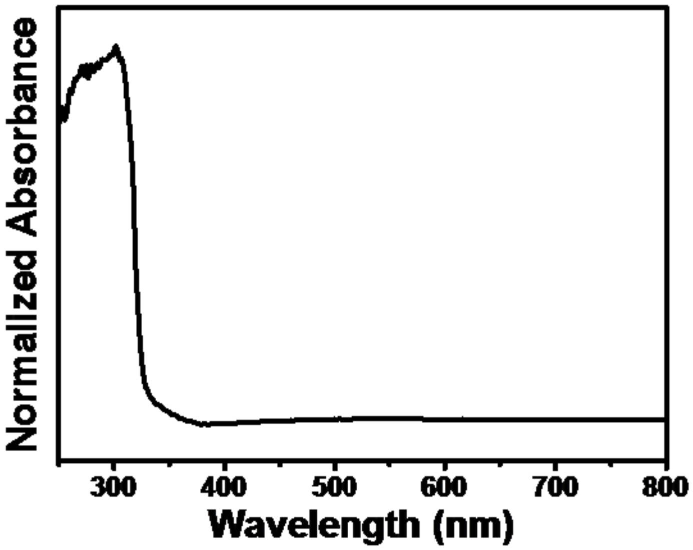

Figure S3. UV-Vis absorption spectrum of the PMMA/TPU film without $CsPbX_3$ PNCs.

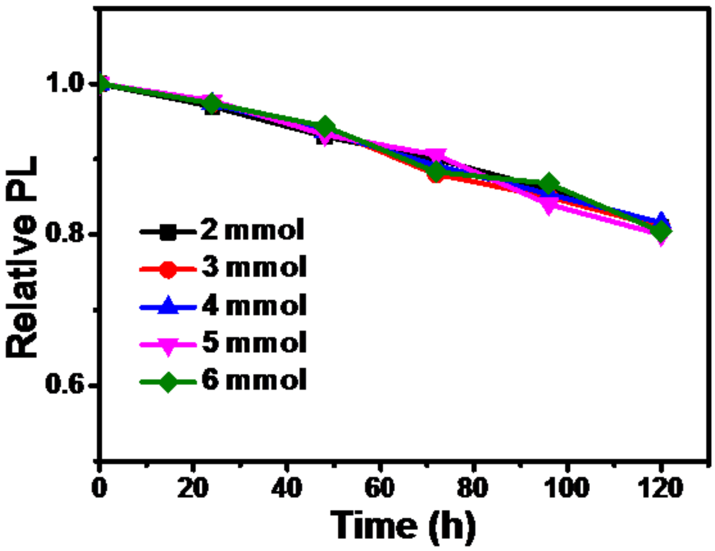

Figure S4. Stability test of $CsPbBr_3$/PMMA/TPU films immersed in water. The molar content of $CsPbBr_3$ was altered whilst keeping the polymer content unchanged.

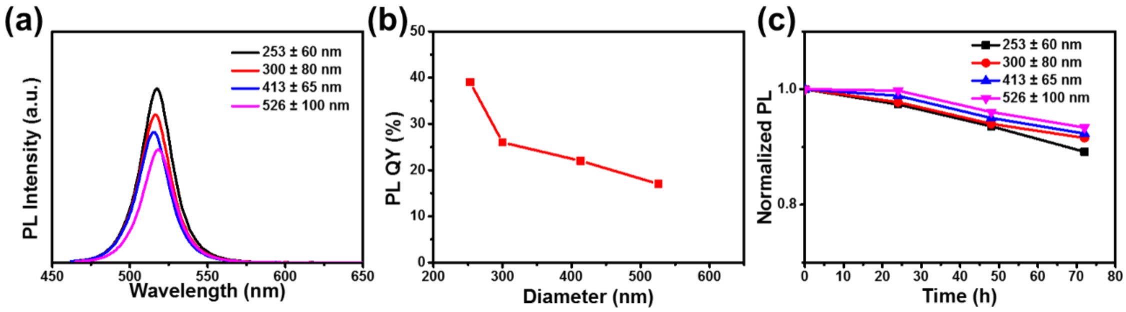

Figure S5. (a) PL spectra, (b) PL QY, and (c) water stability test of $CsPbBr_3$/PMMA/TPU films with different fiber diameters.

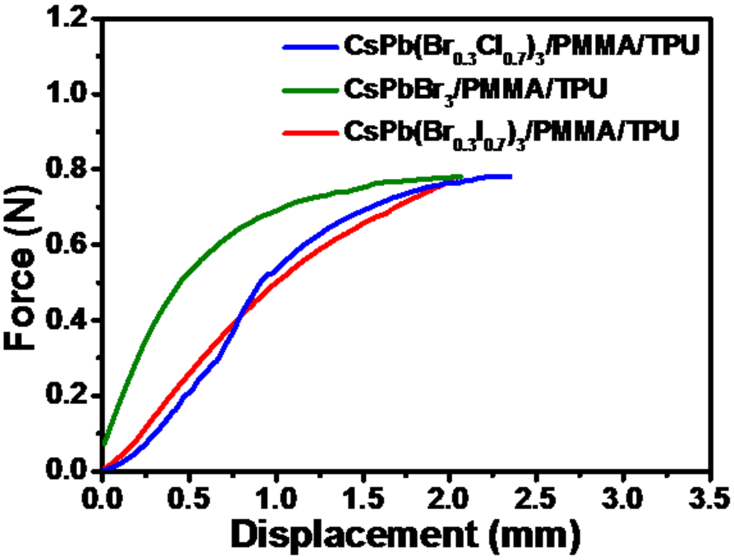

Figure S6. Mechanical strength of $CsPb(Br_{0.3}Cl_{0.7})_3$/PMMA/TPU (blue), $CsPbBr_3$/PMMA/TPU (green) and $CsPb(Br_{0.3}I_{0.7})_3$/PMMA/TPU (red) fibrous films.

Table S1. The fitting results for time-resolved fluorescence decay of $CsPbBr_3$/PMMA/TPU fibrous films and pure $CsPbBr_3$ film

	τ_1 (ns)	B_1 (%)	τ_2 (ns)	B_2 (%)
$CsPbBr_3$/PMMA/TPU	0.54	10.8	21.74	89.2
$CsPbBr_3$	5.15	45.22	20.02	54.78
